# GSK3 regulates the expressions of human and mouse c-Myb via different mechanisms

**DOI:** 10.1186/1747-1028-5-27

**Published:** 2010-11-21

**Authors:** Kyoko Kitagawa, Yojiro Kotake, Yoshihiro Hiramatsu, Ning Liu, Sayuri Suzuki, Satoki Nakamura, Akira Kikuchi, Masatoshi Kitagawa

**Affiliations:** 1 Department of Biochemistry 1, Hamamatsu University School of Medicine, Hamamatsu, Japan; 2 Second Department of Surgery, Hamamatsu University School of Medicine, Hamamatsu, Japan; 3 Third Department of Internal Medicine, Hamamatsu University School of Medicine, Hamamatsu, Japan; 4 Department of Molecular Biology and Biochemistry, Graduate School of Medicine, Osaka University, Suita, Japan

## Abstract

**Background:**

c-Myb is expressed at high levels in immature progenitors of all the hematopoietic lineages. It is associated with the regulation of proliferation, differentiation and survival of erythroid, myeloid and lymphoid cells, but decreases during the terminal differentiation to mature blood cells. The cellular level of c-Myb is controlled by not only transcriptional regulation but also ubiquitin-dependent proteolysis. We recently reported that mouse c-Myb protein is controlled by ubiquitin-dependent degradation by SCF-Fbw7 E3 ligase via glycogen synthase kinase 3 (GSK3)-mediated phosphorylation of Thr-572 in a Cdc4 phosphodegron (CPD)-dependent manner. However, this critical threonine residue is not conserved in human c-Myb. In this study, we investigated whether GSK3 is involved in the regulatory mechanism for human c-Myb expression.

**Results:**

Human c-Myb was degraded by ubiquitin-dependent degradation via SCF-Fbw7. Human Fbw7 ubiquitylated not only human c-Myb but also mouse c-Myb, whereas mouse Fbw7 ubiquitylated mouse c-Myb but not human c-Myb. Human Fbw7 mutants with mutations of arginine residues important for recognition of the CPD still ubiquitylated human c-Myb. These data strongly suggest that human Fbw7 ubiquitylates human c-Myb in a CPD-independent manner. Mutations of the putative GSK3 phosphorylation sites in human c-Myb did not affect the Fbw7-dependent ubiquitylation of human c-Myb. Neither chemical inhibitors nor a siRNA for GSK3β affected the stability of human c-Myb. However, depletion of GSK3β upregulated the transcription of human *c-Myb*, resulting in transcriptional suppression of *γ-globin*, one of the c-Myb target genes.

**Conclusions:**

The present observations suggest that human Fbw7 ubiquitylates human c-Myb in a CPD-independent manner, whereas mouse Fbw7 ubiquitylates human c-Myb in a CPD-dependent manner. Moreover, GSK3 negatively regulates the transcriptional expression of human *c-Myb *but does not promote Fbw7-dependent degradation of human c-Myb protein. Inactivation of GSK3 as well as mutations of Fbw7 may be causes of the enhanced c-Myb expression observed in leukemia cells. We conclude that expression levels of human and mouse c-Myb are regulated via different mechanisms.

## Background

The leucine zipper transcription factor c-Myb is expressed at high levels in immature progenitors of all the hematopoietic lineages, and is essential for fetal liver hematopoiesis, erythroid and myeloid bone marrow colony formation, and T- and B-cell development [1-4]. Moreover, elevated c-Myb expression is associated with hematological malignancies and has been reported in many cases of acute myeloblastic and lymphoblastic leukemias [1,5-7]. The keys to the control of c-Myb protein function are post-transcriptional modifications. The c-Myb protein is phosphorylated by several kinases such as MAPK, Nemo-like kinase (NLK) and glycogen synthase kinase 3 (GSK3) [8-10]. It has been reported that phosphorylation influences the activity and stability of the c-Myb protein [11-17]. The stabilities of many kinds of cellular proteins are often controlled by the ubiquitin proteasome system, a rapid and selective degradation mechanism in cells [18]. A previous study indicated that the stability of c-Myb protein is also regulated by this system. Especially, SCF-type E3 ubiquitin ligases target various important cellular proteins including cell cycle regulators, oncogene and tumor suppressor gene products [19,20]. Recently, we and another group reported that the mouse c-Myb protein levels are regulated by ubiquitin-dependent degradation via SCF-Fbw7 E3 ligase in a phosphorylation-dependent manner [21,22]. Fbw7 targets various proteins, including cyclin E, Notch1, c-Myc, SREBP, c-Jun and SRC-3, for ubiquitylation. These substrates contain a consensus phospho-binding motif for Fbw7, termed the Cdc4 phosphodegron (CPD) [23]. Furthermore, we found that mouse c-Myb Thr-572, which is located in a domain equivalent to the CPD, is phosphorylated by GSK3, thereby allowing recognition by Fbw7 and subsequent promotion of ubiquitin-dependent degradation in the 26 S proteasome [22]. Regarding the regulatory system of human c-Myb, it is unclear whether GSK3 is involved in the control of human c-Myb stability, although we have noticed that human c-Myb is also degraded by Fbw7. In the present study, we analyzed the regions responsible for human c-Myb ubiquitylation by SCF-Fbw7. We also investigated whether repression of GSK3 affected the stability and/or expression of human c-Myb. We found that GSK3 is not involved in human c-Myb protein stability, but plays a role in its transcriptional suppression.

## Materials and methods

### Cell culture

HEK293 and HeLa cells were maintained in Dulbecco's modified Eagle's medium (DMEM) supplemented with 10% fetal bovine serum. K562 cells were maintained in RPMI1640 supplemented with 10% fetal bovine serum.

### Antibodies

The antibodies used in this study were anti-Myc antibody 9B11 (Cell Signaling), anti-Myc antibody 9E10 (Roche), anti-FLAG antibody M2 (Sigma), anti-HA antibody 12CA5 (Roche), anti-c-Myb antibody 1-1 (UPSTATE), anti-Fbw7 antibody H-300 (Santa Cruz) and anti-α-tubulin antibody DM1A (Sigma).

### Plasmids

Complementary DNAs encoding mouse and human c-Myb wild type and their mutants were cloned into pcDNA3.1/Myc-His (Invitrogen) [22]. Expression plasmid of ubiquitin (pCGN-HA-Ub) was previously described [24]. Expression plasmids of pCGN-HA-human-Fbw7 and pcDNA3-FLAG-mouse-Fbw7α were kindly provided by Keiichi Nakayama, Kyushu University. All deletion and point mutants of c-Myb were constructed using standard recombinant DNA techniques.

### Immunoprecipitation

For immunoprecipitation (IP), cell lysates were incubated with 2 μg of antibodies and protein G+ Sepharose 4FF (GE healthcare) at 4°C for 1 h. Immunocomplexes were washed five times with lysis buffer. For double IP, the first immunocomplexes, which were prepared with anti-Myc antibody, were denatured by treatment with SDS sample buffer at 100°C for 8 min. Then ubiquitylated c-Myb was immunoprecipitated again with anti-Myc antibody. Immunoprecipitated samples as well as the original cell lysates (input) were separated by SDS-PAGE and transferred from the gel onto a PVDF membrane (Millipore), followed by immunoblotting (IB). Proteins were visualized using an enhanced chemiluminescence system (Perkin Elmer).

### *In vivo *ubiquitylation assay

All plasmids were transfected into HEK293 cells by the calcium phosphate method. As described in previous reports [22], to induce accumulation of polyubiquitylated c-Myb, cells were treated with the proteasome inhibitor MG132 (20 μM), for 5 h starting at 43 h after transfection and then harvested. Cell lysates were prepared with lysis buffer (50 mM Tris-HCl, pH 7.5, 300 mM NaCl, 0.5% Triton X100, 10 μg/mL each of antipain, pepstatin, E-64, leupeptin, and trypsin inhibitor and 2.5 μg/mL of chymostatin) following IB analysis.

### *In vivo *degradation assay

All plasmids were transfected into HeLa cells with the use of Lipofectamine 2000 (Invitrogen). A total of 24 h after transfection, each transfectant was divided into 5 culture dishes for the chase experiment, and after an additional 24 h or 48 h, cells were treated with 12.5 μg/mL of cycloheximide for the indicated times. Cell lysates were subjected to immunoblotting. The intensity of the bands was quantitated using image analysis software Image Gauge 4.21 (Fujifilm), and the signal intensity of each c-Myb was normalized using the individual levels of α-tubulin.

### GSK3 inhibitor treatment

At 48 h after transfection of the expression plasmids, HeLa cells were cultured in the presence of 60 μM of 2-thio(3-iodobenzyl)-5-(1-pyridyl)-[1,3,4]-oxadiazole (GSK3 inhibitor type II, Calbiochem), 30 μM of 3-(2,4-dichlorophenyl)-4-(1-methyl-1*H*-indol-3-yl)-1*H*-pyrrole-2,5-dione (SB216763, Tocris) or dimethyl sulfoxide (DMSO, vehicle control) for 24 h before treatment of cycloheximide.

### RNA interference

K562 cells were transfected with siRNA oligonucleotides using HiPerFect transfection reagent (Qiagen) according to the manufacturer's protocol. At 48 h after transfection, cells were divided into two and subjected to IB and QRT-PCR analysis. For the degradation assay, 6 h after transfection, each transfectant was divided into 4 culture dishes. After 42 h additional hours, cells were treated with 12.5 μg/mL of cycloheximide for the indicated times. Cell lysates were subjected to IB. The nucleotide sequences of siRNA for GSK3β was 5'- GUAAUCCACCUCUGGCUAC -3' with 3' dTdT overhangs. It encodes the same sequences that it was reported before [25].

### Quantitative real time-PCR (QRT-PCR) analysis

Total RNA was isolated from cells with the use of Isogen (Wako), and subjected to reverse transcription with random hexanucleotide primers and SuperScript Reverse Transcriptase II (Invitrogen). The resulting cDNA was subjected to QRT-PCR using the Rotor-Gene 3000 system (Corbett Research) and the SYBR premix Ex Taq kit (TaKaRa). The amount of the transcripts of interest was normalized against that of 18S rRNA as an internal standard.

### Statistical analysis

Statistical significance of differences was assessed with t-test. A *P *value of < 0.05 was considered statistically significant.

## Results

### Fbw7 promotes the degradation of human as well as mouse c-Myb

**Figure 1:**
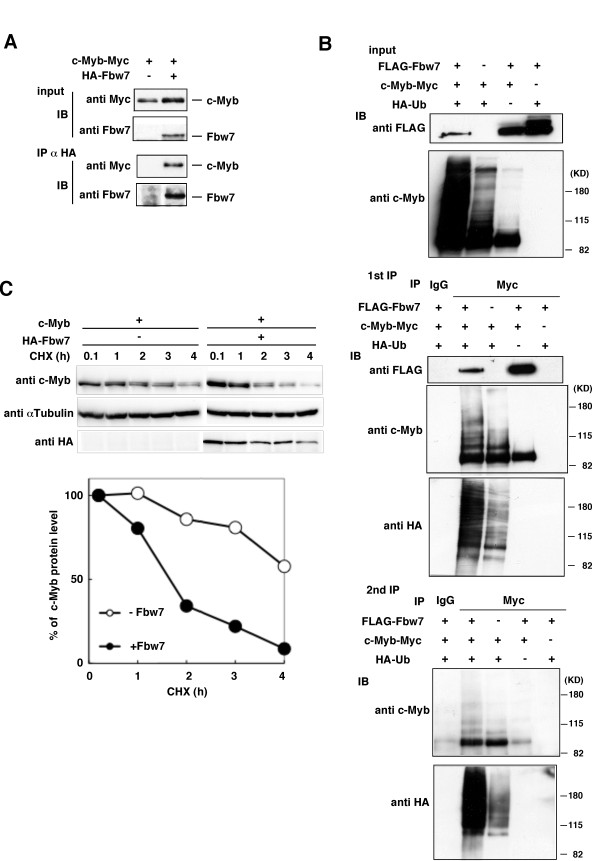
**Fbw7-mediated ubiquitylation and stability control of human c-Myb protein**. (A) Interaction between human c-Myb and human Fbw7. HEK293 cells were transfected with Myc-tagged human c-Myb in the absence or presence of HA-tagged human Fbw7. Cell lysates were subjected to immunoprecipitation (IP) with antibodies against HA, and the resulting precipitates as well as the original cell lysates (input) were subjected to immunoblotting (IB) with antibodies against Myc or Fbw7. (B) HEK293 cells were transfected with expression constructs for Myc-tagged human c-Myb with or without FLAG-tagged human Fbw7 and HA-tagged ubiquitin (Ub) as indicated. The cells were then incubated with MG132. To precisely demonstrate the polyubiquitylation of c-Myb, cell lysates were subjected to double IP with antibodies against Myc or mouse IgG. The resulting precipitates as well as the original cell lysates (input) and the first IP precipitates were subjected to IB with anti-HA, anti-c-Myb or anti-FLAG antibodies. (C) HeLa cells were transfected with human c-Myb in the absence or presence of HA-human Fbw7. For the cycloheximide (CHX) assay, cells were prepared after the indicated times of chase incubation and subjected to IB. The percentages of c-Myb remaining after the various chase times were quantified by image analysis. The immunoblots shown are representative of four independent experiments.

We recently reported that mouse c-Myb is ubiquitylated by SCF-Fbw7 E3 ligase in a phosphorylation-dependent manner and degraded via a ubiquitin proteasome pathway [22]. As shown in Figure 1A, human c-Myb also bound to human Fbw7. To confirm whether the expression of human Fbw7 facilitates ubiquitin conjugation to human c-Myb, we performed *in vivo *ubiquitylation assays for the detection of HA-ubiquitin-modified c-Myb using double immunoprecipitation. Since the first immunoprecipitate may contain c-Myb and its associated proteins, c-Myb was immunoprecipitated as the only component in a second immunoprecipitation after dissociation under denaturing conditions, and subjected to immunoblotting with an anti-HA antibody to evaluate the ubiquitylation of c-Myb itself. The results indicated that Fbw7 promoted the ubiquitylation of human c-Myb (Figure 1B). Furthermore, Fbw7 promoted the degradation of human c-Myb (Figure 1C). These results suggest that SCF-Fbw7 E3 ligase targets c-Myb for ubiquitin-dependent degradation in humans as well as in mice.

### Analysis of the human c-Myb domain required for Fbw7-dependent ubiquitylation

**Figure 2:**
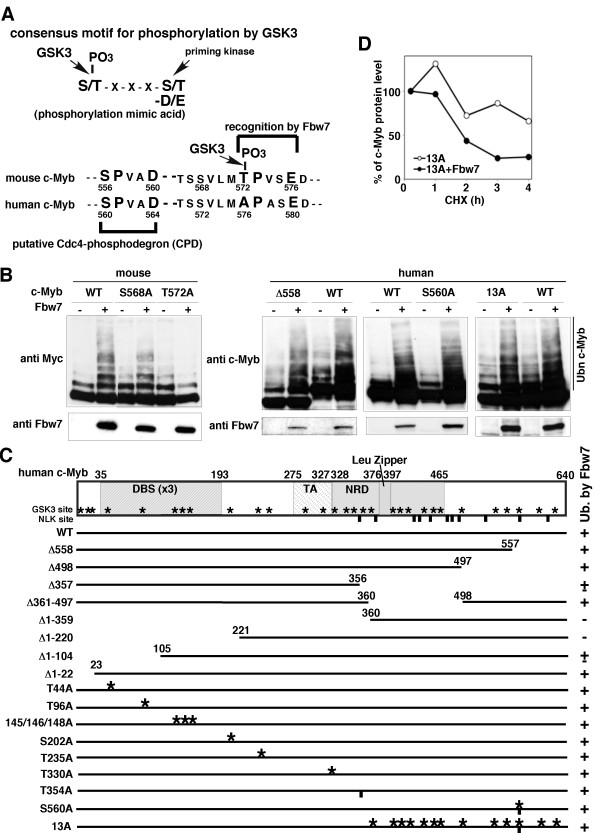
**Fbw7-dependent ubiquitylation of deleted or site-specific amino acid-substituted mutants of human c-Myb**. (A) The consensus motif for phosphorylation by GSK3 is indicated in the upper panel. The human c-Myb sequence is compared with that of mouse c-Myb surrounding Thr-572, which is phosphorylated by GSK3 and then recognized by Fbw7, in the lower panel. Only one putative Cdc4-phosphodegron (CPD) site in human c-Myb is described in parallel. (B) Fbw7-dependent ubiquitylation of mouse and human c-Myb mutants. For analyses of mouse c-Myb ubiquitylation, HA-ubiquitin and Myc-tagged wild-type (WT) or mutant (T568A or T572A) mouse c-Myb in the absence or presence of mouse Fbw7 were transfected into HEK293 cells. For analyses of human c-Myb ubiquitylation, HA-ubiquitin and WT or mutant (Δ558, S560A or 13A) human c-Myb in the absence or presence of human Fbw7 were transfected into HEK293 cells. The transfected cells were then incubated with MG132. Cell lysates were subjected to immunoblotting with an anti-Myc-tag antibody to detect ubiquitylation of the mouse c-Myb protein or with an anti-c-Myb antibody to detect ubiquitylation of the human c-Myb protein. The results for the ubiquitylation of the various mutant human c-Myb proteins by Fbw7 are summarized in (C). (C) Schematic representations of the human c-Myb mutants. The asterisks and vertical bars indicate the putative GSK3 and NLK phosphorylation sites, respectively. DBS, DNA-binding sequence; TA, transactivation domain; Leu Zipper, leucine zipper; NRD, negative regulatory domain. (D) Effects of mutations at the putative GSK3 recognition sites on Fbw7-mediated degradation of c-Myb. HeLa cells were transfected with the human c-Myb mutant 13A in the absence or presence of HA-human Fbw7. For the cycloheximide (CHX) assay, cells were prepared after the indicated times of chase incubation and subjected to immunoblotting. The percentages of c-Myb remaining after the various chase times were quantified by image analysis.

In a previous study, we analyzed the regions of mouse c-Myb responsible for its Fbw7-mediated ubiquitylation and found that Thr-572 in mouse c-Myb is phosphorylated by GSK3 for recognition by Fbw7, which subsequently promotes ubiquitylation in a phosphorylation-dependent manner [22]. However, it is unclear whether GSK3 is also involved in the control of human c-Myb stability. Thr-572 in mouse c-Myb corresponds to a consensus motif for phosphorylation by GSK3 (Figure 2A). Fbw7 selectively recognizes Thr-572 phosphorylated by GSK3 as a putative CPD, and promotes ubiquitin-mediated proteasomal degradation of mouse c-Myb (Figure 2A). This phosphorylation site is substituted by alanine (Ala-576) in human c-Myb, and consequently there is only one putative CPD in the domain from Ser-560 to Asp-564 (Figure 2A). Fbw7-dependent ubiquitylation of mouse c-Myb was eliminated by substituting Thr-572 with alanine (Figure 2B) or by deletion of the C-terminal region, including Thr-572 (data not shown). In contrast, neither deletion of the C-terminal region of human c-Myb, which is equivalent to the critical sequences in mouse c-Myb required for ubiquitylation by Fbw7, nor alanine substitution of Ser-560 affected its Fbw7-dependent ubiquitylation (Figure 2B, C). Moreover, the human c-Myb mutant 13A, which has substitutions of serine/threonine residues to alanine residues in 13 C-terminal putative GSK3 phosphorylation sites, was still ubiquitylated and degraded in an Fbw7-dependent manner (Figure 2B, D). We attempted to identify the regions in human c-Myb responsible for its Fbw7-mediated ubiquitylation. Although we generated diverse deletion mutants of human c-Myb and evaluated them by *in vivo *ubiquitylation assays, only two mutants with deletions in the N-terminal region to amino acid (aa) 220 or 359 (Δ1-220 and Δ1-359, respectively) completely lost Fbw7-dependent ubiquitylation, while two other mutants with a deletion in the N-terminal region to aa 104 (Δ1-104) or loss of 284 aa residues at the C-terminal (Δ357) were markedly affected (Figure 2C). Subsequently, we prepared aa-substituted mutants with mutations of the putative GSK3 phosphorylation sites surrounding the N-terminal region (aa residues 23-220) or C-terminal region (aa residues 356-640). In addition, we substituted Thr-235 or Thr-330 with alanine, because these aa residues are potential GSK3 recognition consensus sequences but rarely conserved between human and mouse c-Myb. All the examined human c-Myb proteins with point mutations retained the capacity for enhanced ubiquitylation by Fbw7 (Figure 2C). Based on these findings, we speculated that the critical domains for Fbw7-dependent ubiquitylation might be contained within aa residues 23-220 and/or 357-640. Although it remains unclear exactly what roles these domains play, they are not likely to be involved in GSK3-dependent phosphorylation.

### Human Fbw7 binds to and ubiquitylates c-Myb in a CPD-independent manner, which is different from mouse Fbw7

**Figure 3:**
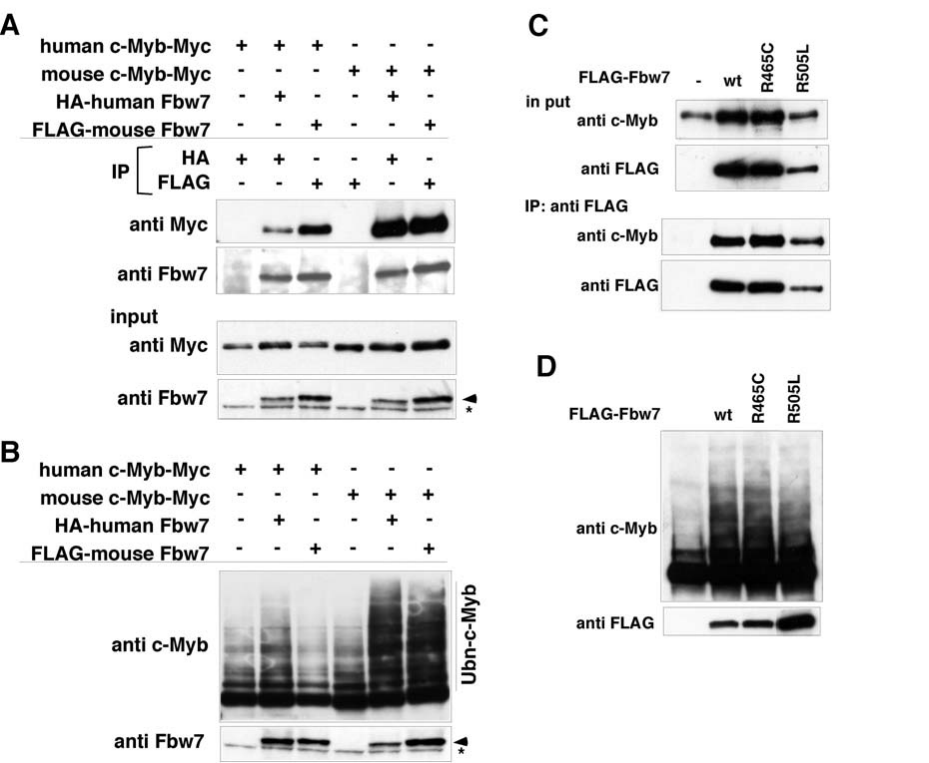
**Human Fbw7 mediates degradation of c-Myb via binding and ubiquitylation, but each system is independent and do not require the functional CPD**. (A) Interaction between human or mouse c-Myb and human or mouse Fbw7. HEK293 cells were transfected with Myc-tagged c-Myb in the absence or presence of Fbw7. Cell lysates were subjected to immunoprecipitation (IP) with antibodies against HA or FLAG, and the resulting precipitates as well as the original cell lysates (input) were subjected to immunoblotting (IB) with antibodies against Myc or Fbw7 which signal corresponded to arrowhead. Asterisk shows non-specific signal. (B) Human or mouse Fbw7-dependent ubiquitylation of mouse and human c-Myb. For analyses of c-Myb ubiquitylation, HA-ubiquitin and Myc-tagged c-Myb in the absence or presence of human or mouse Fbw7 were transfected into HEK293 cells. The transfected cells were then incubated with MG132. Cell lysates were subjected to IB with an anti c-Myb to detect ubiquitylation of the c-Myb protein. Anti Fbw7 antibody was used to detect Fbw7, which signal corresponded to arrowhead. Asterisk shows non-specific signal. (C) Interaction between human c-Myb and human WT or arginine mutant Fbw7. HEK293 cells were transfected with human c-Myb in the absence or presence of FLAG-tagged human Fbw7. Cell lysates were subjected to IP with antibodies against FLAG, and the resulting precipitates as well as the original cell lysates (input) were subjected to IB with antibodies against c-Myb or FLAG. (D) Human Fbw7-dependent but arginine-independent ubiquitylation of human c-Myb. For analyses of human c-Myb ubiquitylation, HA-ubiquitin and human c-Myb in the absence or presence of human WT or arginine mutant Fbw7 were transfected into HEK293 cells. The transfected cells were then incubated with MG132. Cell lysates were subjected to IB with an anti c-Myb or anti FLAG to detect ubiquitylation of human c-Myb protein or Fbw7, respectively.

Although mouse Fbw7 required Thr-572, which is part of the putative CPD motif in mouse c-Myb, human Fbw7 did not require this residue. These observations suggest the presence of different substrate recognition mechanisms between human and mouse Fbw7. We examined the binding and ubiquitylation abilities of human Fbw7 for mouse c-Myb, and of mouse Fbw7 for human c-Myb. We found that Fbw7 bound to c-Myb in all the combinations examined (Figure 3A). Interestingly, human Fbw7 was able to promote the ubiquitylation of both human and mouse c-Myb, whereas mouse Fbw7 was defective in its ability to ubiquitylate human c-Myb (Figure 3B). We further addressed this issue using arginine mutants of human Fbw7, R465C and R505L, which were defective in binding to Notch and promoting its degradation [26]. It is understood that these mutation sites comprise the binding pocket of Fbw7 that permits substrate recognition through contact with phospho-Ser/Thr in the CPD sequence [26]. In this study, we found that both mutants bound to human c-Myb and also had the ability to ubiquitylate human c-Myb as well as wild-type human Fbw7 (Figure 3C, D). These findings suggest that human Fbw7 is able to bind to and ubiquitylate c-Myb in a CPD-independent manner.

### Degradation of human c-Myb occurs in an Fbw7-dependent but a GSK3-independent manner

**Figure 4:**
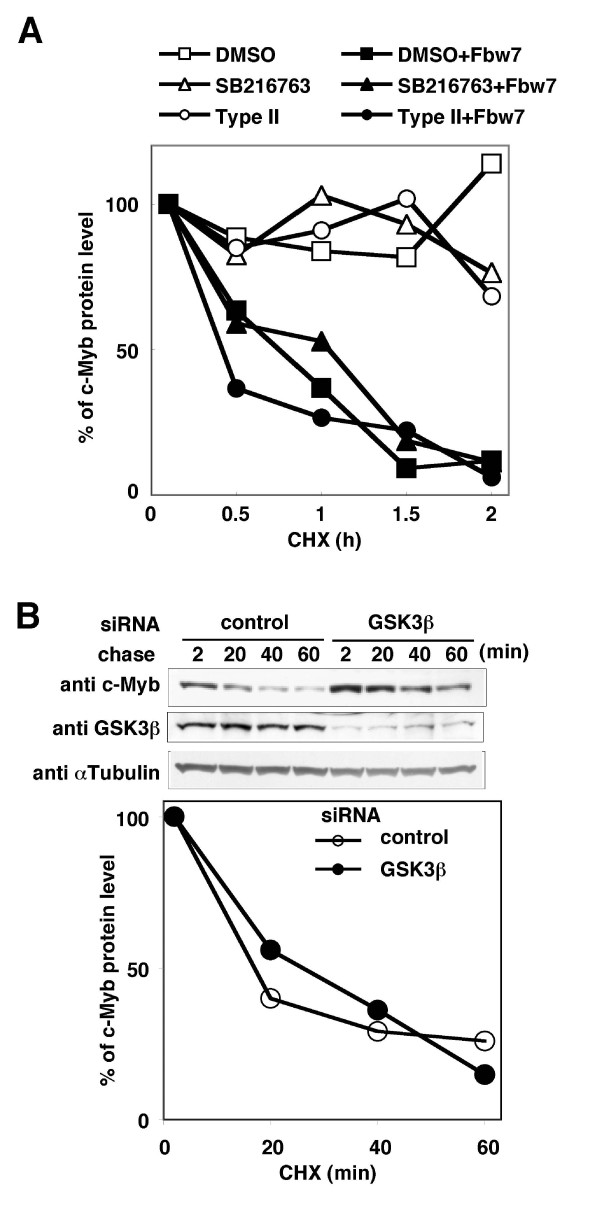
**Effects of GSK3 on human c-Myb degradation**. (A) Effects of GSK3 inhibitors on the stability of human c-Myb. Human c-Myb was transiently expressed with or without human Fbw7 in HeLa cells. The cells were treated with a GSK3 inhibitor, type II or SB216763, or DMSO for 24 h. The cells were then further treated with cycloheximide (CHX) at the indicated times of chase incubation and harvested. (B) Effects of depletion of GSK3β on the stability of human c-Myb protein. K562 cells were transfected with an siRNA for GSK3β or a control siRNA. At 48 h after transfection, the CHX assay was performed.

We modified the cycloheximide (CHX) assay to investigate the necessity of GSK3β activity for the degradation of human c-Myb by Fbw7. GSK3 inhibitors (type II and SB216763) did not inhibited the Fbw7-mediated degradation of human c-Myb (Figure 4A). We also evaluated the impact of SB216763 on K562 cells, and found that it had no effect on the turnover of endogenous human c-Myb (data not shown). To examine whether the abundance of endogenous GSK3 affects the c-Myb stability, we used RNAi to deplete GSK3β in K562 cells and performed the CHX assay. The degradation of endogenous human c-Myb was not affected by depletion of GSK3β (Figure 4B). Taken together, we conclude that the degradation of human c-Myb occurs in an Fbw7-dependent but a GSK3-independent manner. Fbw7 may promote the ubiquitin-dependent degradation of mouse and human c-Myb via two distinct mechanisms.

### Depletion of GSK3β promotes the transcription of human c-Myb and represses the transcription of γ-globin in K562 cells

**Figure 5:**
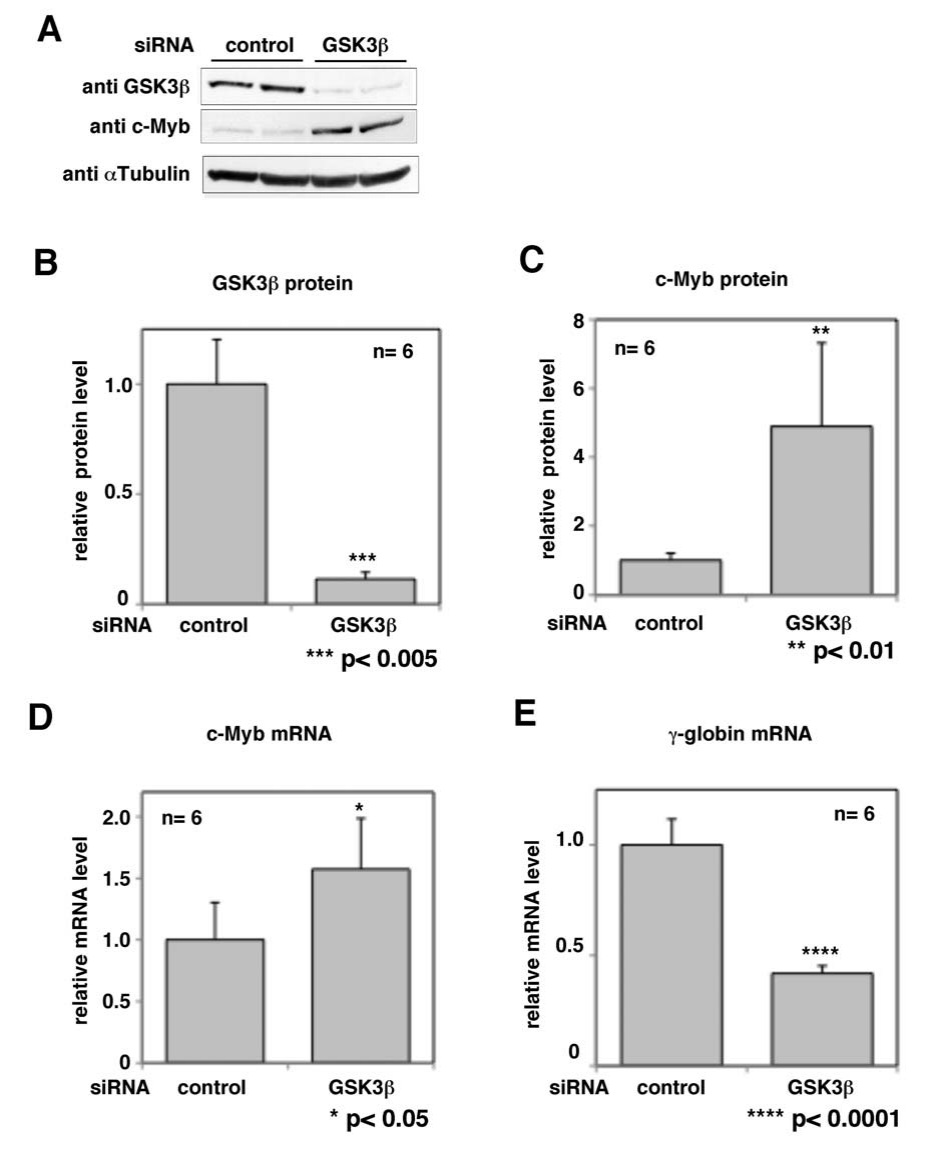
**Depletion of endogenous GSK3β affects the transcriptional expression of human *c-Myb *and promotes the transrepression of *γ-globin *in K562 cells**. K562 cells were transfected with an siRNA for GSK3β or a control siRNA. At 48 h after transfection, the protein levels of GSK3β and c-Myb were analyzed by immunoblotting (A, B, C) and the mRNA levels of c-Myb and γ-globin were measured by quantitative RT-PCR (D, E). The immunoblots in (A) show representative data. Each relative protein or mRNA level was calculated as the mean ± s.d. from six independent experiments.

We previously reported that depletion of Fbw7 mRNA induces the accumulation of c-Myb protein without changing its mRNA level in K562 cells [22]. In chase assays, we noticed that depletion of GSK3β led to the accumulation of human c-Myb protein, but did not affect its stability (Figure 4B). We confirmed again that human c-Myb protein was increased after depletion of GSK3β in K562 cells (Figure 5A, B, C). Furthermore, the c-Myb mRNA level was also significantly increased after depletion of GSK3β in K562 cells (Figure 5D). These findings suggest that GSK3 negatively regulates the transcription of the human *c-Myb *gene. It has been reported that c-Myb inhibits *γ-globin *gene expression in K562 cells [27]. In the present study, we examined the effects of ablation of GSK3β on c-Myb-dependent transcriptional regulation by measuring the level of endogenous *γ-globin *expression by quantitative RT-PCR. The abundance of *γ-*globin transcripts was significantly decreased after depletion of GSK3β (Figure 5E). These results suggest that GSK3 controls the expressions of c-Myb target genes via transcriptional suppression of the *c-Myb *gene in human hematopoietic cells.

## Discussion

GSK3 is one of the coregulators for turnover of several Fbw7 substrates, including cyclin E, c-Jun, Myc, SREBP, Notch, SRC and mouse c-Myb, which have the conserved phospho-epitope known as the CPD [22,26,28-36]. GSK3 phosphorylates the central threonine or serine of the CPD in each substrate. This phosphorylation of the CPD is important for recognition and subsequent degradation by Fbw7. In some cases, the substrates have mutations within their CPDs, resulting in escape from Fbw7-mediated degradation [35,37]. Retroviral Jun proteins contain mutation in their CPD, which result in the acquisition of resistance to Fbw7-dependent degradation [38]. These mutations may contribute to increases in their oncogenic characters. Notch-activated mutations are frequently found in T-cell acute lymphoblastic leukemias (T-ALL). A point mutation at Thr-2512 surrounding the CPD in Notch has been reported as one of the mutation hotspots, and is predicted to abrogate Fbw7 binding [35]. Because the CPDs in Fbw7 substrates play important roles for turnover, it is reasonable for them to be highly conserved across species. Nevertheless, the CPD in mouse c-Myb is not retained in human c-Myb because the equivalent aa residue to Thr-572 in the mouse c-Myb CPD is substituted by an alanine residue in human c-Myb (Figure 2A).

Human Fbw7 ubiquitylated not only human c-Myb but also mouse c-Myb, whereas mouse Fbw7 ubiquitylated mouse c-Myb but not human c-Myb. These findings suggest that human Fbw7 recognizes c-Myb in a different manner from mouse Fbw7. Two arginine mutants (R465C and R505L) of human Fbw7, which are mutated in arginine residues required for recognition of the CPD in Notch, as another substrate of Fbw7 [26], still bound to and ubiquitylated human c-Myb. Therefore, human Fbw7 does not require either Arg-465 or Arg-505 in the β-propeller fold for targeting of c-Myb protein as a substrate. These findings are consistent with the observation that the critical threonine residue (Thr-572) in mouse c-Myb for human Fbw7-dependent ubiquitylation is not conserved in human c-Myb. Although further structural analyses are required to fully resolve the recognition mechanism of human Fbw7 for c-Myb, our data strongly suggest that human Fbw7 ubiquitylates human c-Myb in a CPD-independent manner.

Meanwhile, it has been proposed that Fbw7 binds to and ubiquitylates cyclin E under two kinds of conditions, namely monomeric or dimeric conformations, which depend on the phosphorylation status of cyclin E containing two CPD sites [19,39]. There may be some variety in the substrate recognition mechanism of E3.

Corradini *et al*. [10] suggested that the PI3K/Akt/GSK3β pathway is involved in the stability of human c-Myb, and found increased stabilities of two c-Myb deletion mutants (Δ(358-452) and Δ(389-418)) compared with wild type c-Myb, although the corresponding E3 ligase or phosphorylation sites were not identified. More phosphorylation sites and/or multiple kinases may be needed for the degradation of human c-Myb. Kanei-Ishii *et al*. [21] reported that the mouse c-Myb/Fbxw7 interaction was enhanced by NLK, whose recognition site (S/T-P) is also part of a consensus motif for GSK3 phosphorylation. We cannot exclude the possibility that NLK may partially contribute to the recognition of human c-Myb by Fbw7, although substitutions of the S/T-P sites to alanine or C-terminal deletion mutants that lacked some putative NLK sites did not influence the ubiquitylation ability of Fbw7 (Figure 2C, T354A, S560A, 13A, Δ498 and Δ361-497).

Elevated c-Myb expression has been reported in many cases of acute myeloblastic and lymphoblastic leukemias [6,7]. There are several possible mechanisms underlying such increases. The first is gene amplification of c-Myb, the second is enhancement of c-Myb protein stability caused by a defect in Fbw7 resulting from a gene mutation, and the third is facilitation of gene transcription of c-Myb. Practically, there have been some reports of c-Myb gene amplification cases and frequent Fbw7 gene mutations in T-ALL [40,35]. In this study, we found that GSK3 repressed the transcription of human c-Myb mRNA. GSK3 participates in cell cycle regulation and is a downstream target of the PI3K/Akt pathway, which inhibits GSK3 through phosphorylation of Ser-9. The PI3K/Akt pathway is activated by several kinds of growth factors. Therefore, growth factor stimuli suppress GSK3 activity via PI3K/Akt, and this may lead to enhance c-Myb expression. This mechanism is supported by our findings that depletion of GSK3 augmented *c-Myb *expression and repressed *γ-globin *expression. Alternatively, gene amplifications of *PI3K *have been reported in human cancer [41]. It is possible that inactivation of GSK3 via accelerated PI3K activity leads to the induction of c-Myb transcription in leukemia. Kohmura *et al*. [42] reported that the p38 MAPK and ERK pathways are involved in the differentiation of K562 cells induced by STI571, a specific tyrosine kinase inhibitor of Abl kinase. The expression level of c-myb mRNA was clearly downregulated in K562 cells after incubation with STI571. Their findings and the present results suggest that kinase activity regulates cellular differentiation through the transcriptional repression of human c-Myb.

To investigate whether GSK3 also regulates the transcription of mouse c-Myb, we examined its influence on mouse c-Myb expression using GSK3 inhibitors. The results revealed that GSK inhibitors did not affect the transcriptional regulation of mouse c-Myb (data not shown). Moreover, we tried to confirm these results by GSK3 knockdown using mouse cell line (M-1), in which c-Myb expression was detected. However, we were unable to achieve sufficient knockdown of GSK3 for such evaluations in these cells. The species-specificity of the Fbw7 operating mechanism does appear to be an intriguing issue. However, the transcriptional regulation of c-Myb by GSK3 cannot presently be concluded to be a human-specific event.

c-Myb is abundantly expressed in immature erythroid progenitor cells, and is reduced as the cells mature. It has been observed that GATA-1, one of the erythroid lineage-specific transcriptional factors, represses c-Myb transcription through GATA-1-binding sites in the c-Myb promoter during erythroid differentiation [43]. GATA-1 activity is regulated by post-translational systems, which include a nuclear translocation process [44]. Hyperphosphorylated GATA-1 protein is preferentially found in the nucleus and has an enhanced DNA-binding capacity [44,45]. Phosphorylation of GATA-1 may also have other functions, such as modulation of the binding site preferences or interactions with other transcriptional regulators. GATA-1 is phosphorylated *in vivo *on seven serine residues [46]. Towatari *et al*. [47] identified MAPK as one of the kinases that acts on GATA-1, and further identified Ser-26 and Ser-178 as the phosphorylation sites for MAPK. Zhao *et al*. [48] identified the PI3K/AKT signaling pathway as a mediator of erythropoietin-induced phosphorylation of GATA-1 at Ser-310, resulting in enhancement of its transcriptional activity. They also found that the effects of AKT during the program of erythroid maturation were not limited to phosphorylation of Ser-310, and described that AKT or other PI3K-dependent kinases may phosphorylate additional sites on GATA-1. It is not yet known whether GSK3 participates in the control of GATA-1 activity. Further studies are required to elucidate the roles of kinases in the modulation of GATA-1.

Conditional inactivation of Fbw7 leads to the development of lymphoma and T-ALL in mice [49-51]. It appears that Fbw7 E3 ligase preferentially targets the regulators of hematopoiesis, such as c-Myc and Notch, which interact with Fbw7 in the GSK3-mediated phosphorylated form. Therefore, GSK3 as well as Fbw7 is important controller of the c-Myb, c-Myc and Notch protein levels for appropriate and sequential maturation of hematopoietic cells. Although it has not yet been discussed whether the GSK3 activity changes in patients with leukemia, attenuation of GSK3 activity may have a pleiotropic influence on leukemia progression through the transcriptional and post-transcriptional regulation of c-Myb.

## Conclusions

In the mouse c-Myb protein, GSK3 phosphorylates Thr-572, leading to recognition by Fbw7 for the promotion of ubiquitin-dependent degradation. Fbw7 also promotes the ubiquitylation and proteasome-mediated degradation of human c-Myb, while GSK3 is not involved. Alternatively, GSK3 negatively regulates the transcriptional expression of human *c-Myb *to enhance the transcription of *γ-globin*, a target gene of c-Myb. Therefore, GSK3 regulates the expressions of human and mouse c-Myb via different mechanisms. Inactivation of GSK3 as well as mutations of Fbw7 may be involved in the elevated c-Myb expression observed with human leukemia development.

## Competing interests

The authors declare that they have no competing interests.

## Authors' contributions

KK and MK conceived of the study and drafted the manuscript. KK and YH performed the experiments. YK, YH, SS, NL, SN and AK participated in its design and coordination. All authors read and approved the final manuscript.
